# Adverse drug reactions associated with six commonly used antiepileptic drugs in southern China from 2003 to 2015

**DOI:** 10.1186/s40360-019-0285-y

**Published:** 2019-01-14

**Authors:** Yanru Du, Jiahe Lin, Jingzan Shen, Siqi Ding, Mengqian Ye, Li Wang, Yi Wang, Xinshi Wang, Niange Xia, Rongyuan Zheng, Hong Chen, Huiqin Xu

**Affiliations:** 1 Department of Neurology, The First Affiliated Hospital of Wenzhou Medical University, Shangcai Village, Ouhai District, Wenzhou, Zhejiang Province People’s Republic of China; 2 Department of Preventive Medicine, School of Public Health and Management, Wenzhou Medical University, Wenzhou, Zhejiang Province People’s Republic of China; 3 Department of Psychiatry, The First Affiliated Hospital of Wenzhou Medical University, Wenzhou, Zhejiang Province People’s Republic of China

**Keywords:** Adverse drug reactions, Antiepileptic drug, Severe adverse reactions, China

## Abstract

**Background:**

This active, open observational study aimed to investigate adverse drug reactions (ADRs) associated with six commonly used antiepileptic drugs (AEDs) in southern Chinese outpatients with epilepsy from 2003 to 2015.

**Methods:**

The Wenzhou Epilepsy Follow-Up Registry Database (WEFURD) was established by a single epilepsy center in China in January 2003 to record AED efficacy and the associated ADRs by registered outpatients diagnosed with epilepsy. We reviewed the data of outpatients who had only taken one or more of six commonly used AEDs, namely, carbamazepine (CBZ), valproate (VPA), lamotrigine (LTG), oxcarbazepine (OXC), topiramate (TPM) and levetiracetam (LEV), and were registered in the WEFURD between 2003 and 2015. We evaluated the ADRs caused by the single or combined use of the above six specific AEDs based on the WHO-UMC scale. The data of the ADRs were categorized by age, sex, number of AEDs related to ADRs, medications, seriousness of ADRs, causality levels of the WHO-UMC scale and system organ class (SOC). The unit of analysis was one ADR.

**Results:**

A total of 3069 epilepsy outpatients (1807 outpatients with 5049 eligible ADRs and 1262 outpatients without ADRs) were included. The overall ADR rate was 58.88% (1807/3069). An average of 2.79 ADRs (5049/1807) occurred per patient with an ADR; 53.8% of the 5049 ADRs were recorded in females, and 50.4% were caused by monotherapy. Of the ADRs, 10.6% (537/5049) were severe adverse reactions (SARs), including 34 serious adverse effects (SAEs). The SAR rates caused by one, two and three or more AEDs were 9.9, 10.0 and 19.6%, respectively (*p* <  0.001). Eighteen SOC categories were identified, and the top three were psychiatric disorders (1633/5049, 32.3%), neurological disorders (1222/5049, 24.2%) and gastrointestinal disorders (564/5049, 11.2%). Of the 537 SARs, skin and appendage disorders accounted for 24.4% (131/537). Among the 34 SAEs, serious allergies, fetal malformations, renal calculus and pancreatitis accounted for the majority.

**Conclusions:**

Our findings suggest that clinicians should pay attention to psychiatric ADRs and be alert for SARs, especially when three or more AEDs are used together. Moreover, active surveillance might provide another method of pharmacovigilance in China.

**Electronic supplementary material:**

The online version of this article (10.1186/s40360-019-0285-y) contains supplementary material, which is available to authorized users.

## Background

Adverse drug reactions (ADRs) are a major public health concern [1]. In recent years, the most widely used antiepileptic drugs (AEDs) in our epilepsy center included carbamazepine (CBZ), valproate (VPA), lamotrigine (LTG), oxcarbazepine (OXC), topiramate (TPM) and levetiracetam (LEV). ADRs to AEDs may lead to treatment failure, withdrawal or death [2]. In 2007, an unblinded randomized controlled trial (RCT) was conducted to assess the effectiveness and safety of CBZ, LTG, OXC, or TPM for the treatment of partial epilepsy. The results of this study showed that approximately 50% of patients reported adverse events [3]. Meta-analyses in the literature showed that AEDs had aggression as a side effect [4], and the behavioral side-effect profiles of AEDs should be considered when choosing optimal AEDs [5]. Furthermore, a recent article reported that the use of aromatic AEDs, e.g., CBZ, OXC and LTG, was more frequently associated with drug hypersensitivity [6]. Attention should also be paid to safety concerns regarding the use of AEDs during pregnancy and breastfeeding [7]. RCTs have improved the quality and reliability of drug evaluation studies, but they have not provided specific clinical safety data [8]. However, for various ethical, statistical and logistical reasons, it is extremely difficult to organize structured clinical studies that are likely to provide substantial data on ADRs [9]. In China, the safety monitoring of AEDs mainly relies on the implementation of the national reporting system of ADRs, which is a spontaneous reporting (SR) system. The reporting of ADRs to the China Food and Drug Administration (CFDA) has been the duty of healthcare professionals. In the SR system, however, the problems of underreporting and poor-quality data are still difficult to solve. Active monitoring from clinicians can compensate for the deficiencies of the SR system, but such research is relatively lacking.

To fill this gap, we conducted a clinical, active, open observational study to investigate the ADRs associated with six commonly used AEDs in epilepsy outpatients in southern China from 2003 to 2015.

## Methods

### Data source

The Epilepsy Long-term Follow Up Registry Study (ELFURS) was a single-center, prospective and observational study of epilepsy outpatients in China. The ELFURS was conducted at the epilepsy center in the First Affiliated Hospital of Wenzhou Medical University (FAHWMU) in January 2003 [10]. The study was approved by the clinical research ethics committee of FAHWMU and registered in the World Health Organization (WHO) Registry Network (registration number: ChiCTR-OCH-14004616). The Wenzhou Epilepsy Follow-Up Registry Database (WEFURD) was established simultaneously to record, save, and process the registry data, which included the demographic information of the epileptic outpatients, the use of AEDs, the efficacy of the AEDs and the AED-related ADRs [10]. An ADR, as defined by the WHO in 1972, is “a response to a drug that is noxious and unintended and occurs at doses normally used in man for the prophylaxis, diagnosis or therapy of disease, or for modification of physiological function” [11]. ADR data were prospectively collected at every visit by questionnaire (every 1–3 months), and relevant information was updated in the following visits. The WEFURD has been maintained by trained two researchers and is the largest epilepsy database in southern China. By January 2018, the WEFURD enrolled 4563 epilepsy outpatients with regular follow-up and included 6259 records of ADRs.

### Study protocol

In this study, we reviewed the data of outpatients who only took one or more of six commonly used AEDs (CBZ, VPA, LTG, OXC, TPM and LEV) and were registered in the WEFURD between 2003 and 2015. We evaluated the ADRs caused by single or combined use of the above six specific AEDs based on the WHO-UMC scale [12]. ADRs that were not caused by these six specific AEDs were excluded. ADRs that occurred when these six AEDs were combined with the other AEDs were also excluded. Finally, 1807 epilepsy outpatients with 5049 eligible ADRs and 1262 epilepsy outpatients without ADRs were included in this study. The two groups (epilepsy outpatients with and without ADRs) were compared, and the data of the 5049 ADRs were categorized by age, sex, number of AEDs related to ADRs, medications, severity of ADRs, causality levels of the WHO-UMC scale [12] and system organ class (SOC) according to the WHO Adverse Reactions Terminology (WHOART) [13]. The unit of analysis was one ADR. The preferred terms (PTs) of the WHOART [13] were used to describe and quantify the ADRs.

### Validation and classification of ADRs by the WHO-UMC scale

The WHO-UMC criteria [12] were employed to classify the causality levels as certain, probable/likely, possible, unlikely, conditional/unclassified, and unassessable/unclassifiable. Prior to assessment, 12 members of the project received unified training. The causality assessment of an ADR was performed independently by two physicians, and this work was completed by eight physicians. The other two physicians checked the consistency of the assessment results. For ADRs with inconsistent assessment results, the two epileptologists (Huiqin Xu and Rongyuan Zheng) re-evaluated the results together. The ADRs that were still controversial after the reassessment process were discussed by all of the team members. We minimized bias from the evaluators through unified training, verification and discussion. The levels of unlikely and unassessable/unclassifiable were excluded in this study.

### Classification of ADRs by SOC and severity

The 5049 ADRs were classified according to the SOCs in the WHOART [13]. Serious adverse effects (SAEs) were defined as those that are lethal or life threatening, require hospital admission or prolongation of an existing hospital stay, result in persistent or significant disability/incapacity, or are cancers, congenital anomalies, birth defects, or other medically important conditions [14]. The term “severe” is often used to describe the intensity (severity) of a medical event or an adverse reaction, as in the grading “mild”, “moderate”, and “severe”; thus, a severe skin reaction is not necessarily serious [14]. “Serious” and “severe” are different; SAEs are severe, but severe adverse reactions (SARs) are not necessarily serious. To improve the accuracy of the term “severe” in practical applications, SARs in this study were defined as adverse reactions requiring withdrawal of the suspected drugs and symptomatic treatment, regardless of whether the suspected drugs were used in mono- or polytherapy.

### Classification of SARs by drugs

The SARs were analyzed according to the suspected AEDs. In addition, the dosage/defined daily dose (DDD) [15] was used to represent the total daily dose of the suspected drugs [16]. The DDDs of the six drugs were as follows: 300 mg TPM and LTG, 1000 mg CBZ and OXC, and 1500 mg VPA and LEV [15]. If ADRs occurred when AEDs were used in polytherapy, the total daily dose of all drugs was calculated as the sum of the dosage/DDD for each drug. For example, if dizziness occurred while taking 300 mg/day CBZ and 1000 mg/day LEV, the total daily dose of the two drugs was calculated as 300/1000 + 1000/1500 = 0.97.

### Statistics

Categorical/qualitative data are presented as numbers (percentages), and nonnormally distributed quantitative data are shown as the medians (ranges). The Pearson chi-square and Mann-Whitney U tests were used, as appropriate. A two-sided *p* <  0.05 was considered significant. A Bonferroni correction was applied for the multiple comparisons, and the *p*-values were adjusted according to the number of tests (*n* = 3 for the number of AEDs received and *n* = 3 for the number of AEDs related to the ADR). A corrected *p* = 0.0167 (0.05/3) was considered significant for the number of AEDs received and the number of AEDs related to the ADR. All statistical analyses were conducted using IBM SPSS Statistics 21.0 software (IBM Corporation, NY, USA).

## Results

### Characteristics of epilepsy outpatients with and without ADRs

A total of 3069 epilepsy outpatients (1807 epilepsy outpatients with 5049 eligible ADRs and 1262 epilepsy outpatients without ADRs) were included. The overall ADR rate was 58.88% (1807/3069). We compared 1807 epilepsy outpatients with ADRs and 1262 epilepsy outpatients without ADRs (Table 1). There was no significant difference in age between the two groups, regardless of the age at last visit or at epilepsy diagnosis. Compared with epilepsy outpatients without ADRs, females comprised a higher percentage of epilepsy outpatients with ADRs (49.4% vs. 38.9%, *p* <  0.001).

**Table 1:** Characteristics of epilepsy outpatients with and without ADRs (total = 3069)

Variable	Epilepsy outpatients with ADRs (*N* = 1807)	Epilepsy outpatients without ADRs (*N* = 1262)	*p*-value
Age at last visit, years (n, %)			0.156^a^
≤ 12	395 (21.9)	287 (22.7)	
13–18	499 (27.6)	323 (25.6)	
19–45	783 (43.3)	536 (42.5)	
> 45	130 (7.2)	116 (9.2)	
Age at epilepsy diagnosis, years (median, (range))	18.2 (0.17–86.0)	18.3 (0.1–81.3)	0.770^b^
Sex (n, %)			< 0.001^a^
Female	893 (49.4)	491 (38.9)	
Male	914 (50.6)	771 (61.1)	
Comorbidity (n, %)			< 0.001^a^
Yes	374 (20.7)	180 (14.3)	
No	1433 (79.3)	1082 (85.7)	
Number of AEDs received (n, %)^c^			< 0.001^a^
One	489 (27.1)	756 (59.9)	
Two	645 (35.7)	371 (29.4)	
Three+	673 (37.2)	135 (10.7)	

*ADRs* adverse drug reactions, *AEDs* antiepileptic drugs

^a^Pearson chi-square test

^b^Mann-Whitney U test

^c^Multiple comparison, *p* = 0.05/3 = 0.0167 after Bonferroni correction. Two vs. one *p* <  0.001; three+ vs. one *p* <  0.001; three+ vs. two *p* <  0.001

### General information on ADRs

An average of 2.79 ADRs (5049/1807) occurred per patient with an ADR. In total, 53.8% of the ADRs were recorded in females and 46.2% in males, while 50.4% were caused by a single AED (Table 2).

**Table 2:** General profiles of non-SARs and SARs in this study

Variable	Total (*n* = 5049)	non-SARs^e^ (*n* = 4512)	SARs^e^ (*n* = 537)	*p*-value
Age at ADR occurrence, years (median, (range))	28.0 (7.0–89.0)	28.0 (7.0–89.0)	28.0 (8.0–75.0)	0.349^b^
Sex (n, %)				0.006^a^
Female	2717 (53.8)	2398 (53.1)	319 (59.4)	
Male	2332 (46.2)	2114 (46.9)	218 (40.6)	
Number of AEDs related to the ADR^c^				< 0.001^a^
One (n, %)	2546 (50.4)	2294 (90.1)	252 (9.9)	
Two (n, %)	2140 (42.4)	1926 (90.0)	214 (10.0)	
Three+ (n, %)	363 (7.2)	292 (80.4)	71 (19.6)	
AEDs^d^				
CBZ (n, %)	1138 (22.5)	1000 (87.9)	138 (12.1)	0.064^a^
VPA (n, %)	2493 (49.4)	2228 (89.4)	265 (10.6)	0.989^a^
LTG (n, %)	1149 (22.8)	982 (85.5)	167 (14.5)	< 0.001^a^
OXC (n, %)	1226 (24.3)	1109 (90.5)	117 (9.5)	0.154^a^
TPM (n, %)	1186 (23.5)	1050 (88.5)	136 (11.5)	0.288^a^
LEV (n, %)	742 (14.7)	664 (89.5)	78 (10.5)	0.906^a^
Dosage/DDD (median, (range))	0.90 (0.07–3.50)	0.90 (0.07–3.50)	0.67 (0.08–2.13)	< 0.001^b^
Dosage/DDD groups				
≤ 0.50 (n, %)	1061 (21.0)	907 (85.5)	154 (14.5)	< 0.001^a^
0.50–1.00 (n, %)	2309 (45.7)	2074 (89.8)	235 (10.2)	0.332^a^
1.00–1.50 (n, %)	1088 (21.6)	983 (90.3)	105 (9.7)	0.234^a^
1.50–2.00 (n, %)	473 (9.4)	433 (91.5)	40 (8.5)	0.106^a^
> 2.00 (n, %)	118 (2.3)	115 (97.5)	3 (2.5)	0.004^a^
Level of causality assessment (n, %)				
Certain	550 (10.9)	300 (54.5)	250 (45.5)	< 0.001^a^
Probable/Likely	796 (15.8)	510 (64.1)	286 35.9)	< 0.001^a^
Possible	3509 (69.5)	3508 (99.9)	1 (0.1)	< 0.001^a^
Conditional/Unclassified	194 (3.8)	194 (100)	0 (0)	< 0.001^a^

*ADRs* adverse drug reactions, *AEDs* antiepileptic drugs, *SARs* severe adverse reactions, *DDD* defined daily dose

^a^Pearson chi-square test

^b^Mann-Whitney U test

^c^Multiple comparison, *p* = 0.05/3 = 0.0167 after Bonferroni correction. Two vs. one *p* < 0.907; three+ vs. one *p* < 0.001; three+ vs. two *p* < 0.001

^d^The sum of the six AEDs was not equal to 5049 due to half of the ADRs being caused by two or three+ AEDs

^e^SARs refers to those ADRs requiring withdrawal of the suspected drugs and symptomatic treatment, regardless of whether the suspected drugs were used in mono- or polytherapy

Of the 5049 ADRs, 537 were SARs. The general profiles of non-SARs and SARs in this study is displayed in Table 2. The rates of SARs caused by one, two and three or more AEDs were 9.9, 10.0 and 19.6%, respectively (*p* <  0.001). The results showed that the rate of SARs caused by three or more AEDs was significantly higher than the rate of SARs caused by one or two AEDs.

### ADRs and SARs by SOC

Table 3 shows the distribution of the 5049 ADRs and 537 SARs by SOC. Altogether, 18 SOC categories were identified in this study; the top three were psychiatric disorders (1633/5049, 32.3% of the total ADRs), neurological disorders (1222/5049, 24.2% of the total ADRs) and gastrointestinal disorders (564/5049, 11.2% of the total ADRs). Regardless of whether the ADRs were caused by one, two, or three or more AEDs, the most commonly involved categories remained the same. There were 537 SARs (10.6% of the total ADRs). Among these, the most common category was skin and appendage disorders, accounting for 24.4% (131/537).

**Table 3:** Number of adverse drug reactions and severe adverse reactions stratified by system organ class

System organ class (descending order)	All	Number of AEDs related to the ADR		
		One AEDs	Two AEDs	More than three AEDs
	Number (severe^a^)	Number (severe^a^)	Number (severe^a^)	Number (severe^a^)
Psychiatric disorders	1633 (84)	813 (29)	710 (39)	110 (16)
Neurological disorders	1222 (93)	630 (46)	500 (37)	92 (10)
Gastrointestinal disorders	564 (68)	244 (26)	280 (31)	40 (11)
Skin and appendage disorders	341 (131)	183 (67)	132 (50)	26 (14)
Body as a whole - general disorders	260 (19)	137 (10)	108 (5)	15 (4)
Metabolic and nutritional disorders	257 (19)	131 (8)	107 (8)	19 (3)
Liver and biliary disorders	236 (33)	135 (17)	84 (12)	17 (4)
Vision disorders	88 (6)	28 (2)	45 (3)	15 (1)
Reproductive disorders	88 (16)	48 (9)	36 (4)	4 (3)
Blood disorders	81 (25)	32 (15)	37 (9)	12 (1)
Musculoskeletal disorders	65 (11)	39 (5)	23 (4)	3 (2)
Urinary tract disorders	55 (9)	30 (5)	23 (3)	2 (1)
Hearing, vestibular and special senses disorders	49 (2)	32 (1)	16 (1)	1 (0)
Cardiovascular disorders	42 (2)	26 (2)	13 (0)	3 (0)
Vascular, bleeding and clotting disorders	36 (2)	19 (1)	16 (1)	1 (0)
Respiratory disorders	15 (4)	9 (1)	4 (2)	2 (1)
Immune disorders and infections	13 (9)	8 (6)	4 (3)	1 (0)
Congenital disorders	4 (4)	2 (2)	2 (2)	0 (0)
Total	5049 (537)	2546 (252)	2140 (214)	363 (71)

*ADRs* adverse drug reactions, *AEDs* antiepileptic drugs, *SARs* severe adverse reactions

^a^SARs refers to those ADRs requiring withdrawal of the suspected drugs and symptomatic treatment, regardless of whether the suspected drugs were used in mono- or polytherapy

### SARs by drugs

Detailed information on the 537 SARs (including 34 SAEs) stratified by drug is shown in Additional file 1: Table S1. The top 10 most frequent SARs were rash (mainly caused by CBZ, LTG and OXC), nausea and vomiting, dizziness, increased levels of hepatic enzymes, leukopenia, somnolence, tremor, amnesia, hypoesthesia and pruritus. Only 34 SARs were SAEs, of which the following 17 were caused by a single drug: CBZ (*n* = 2, 2 drug hypersensitivity syndrome; DHS), LTG (*n* = 2, 1 erythema multiforme and 1 multiple malformations), OXC (*n* = 1, 1 DHS), TPM (*n* = 9, 5 renal calculus, 1 amnesia, 1 congenital spinal tumor, 1 cognitive disorder, 1 exfoliative dermatitis), and VPA (*n* = 3, 1 thrombocytopenia, 1 abnormal vision, 1 DHS). The other 17 SAEs were observed with combination therapy (Additional file 1: Table S1).

## Discussion

ADRs contribute significantly to patient morbidity and mortality worldwide. Both safety and tolerability are determining factors in the selection of an appropriate AED [17]. There are few observational studies on the safety of AEDs in epilepsy patients in China. In the present study, the two groups (1807 epilepsy outpatients with ADRs and 1262 epilepsy outpatients without ADRs) were compared and the data of the 5049 ADRs, which were caused by single or combinations of six commonly used AEDs, were analyzed. The results of our study provide a reference for clinicians to safely use AEDs.

Our results showed that the overall ADR rate was 58.88%, which is slightly higher than the rates reported in other studies. In studies by Marson AG et al. [3] and Androsova G et al. [17], the ADR rates were approximately 50 and 47.6%, respectively. Of the 5049 ADRs, 53.8% occurred in females. A total of 18 SOC categories were identified, and the most common ADR categories, whether resulting from a single drug or multiple drugs, were psychiatric, neurological and gastrointestinal disorders. There were 537 SARs, 34 of which were SAEs, with the majority being serious allergies, fetal malformations, renal calculus, and pancreatitis. Our study confirmed that SAEs are severe but that SARs are not necessarily serious. This distinction plays a vital role in the precise use of the terms “serious” and “severe” by clinicians.

In this study, less than 2% of the ADRs occurred in children. This low percentage may have been due to selection bias, as 95% of the WEFURD patients were adults. This study showed that ADRs were more frequent in females, and some studies on AED safety have reported similar findings [18]. Differences in ADRs due to sex may be attributed to a greater number of ADRs in females and intolerable ADRs in females. There were approximately three ADRs (2717/893) per female and 2.5 (2332/914) per male. Females exhibited more SARs than non-SARs (59.4% vs. 53.1%, *p* = 0.006). Of the 18 SOC categories, most ADRs were categorized as psychiatric, neurological and gastrointestinal disorders, which were also the SOCs in which most ADRs to nervous system medications were found [19, 20]. As the central nervous system (CNS) is the major action site for AEDs [21, 22], it is not surprising that the CNS was the system most commonly affected by AED-related ADRs [20, 23]. However, there were more ADRs categorized as psychiatric disorders, and ADRs with a frequency > 5% of the psychiatric ADRs presented as follows: amnesia (*n* = 492, 30.13%); somnolence (*n* = 304, 18.62%); insomnia (*n* = 221, 13.53%); anorexia (*n* = 197, 12.06%); and irritability (*n* = 161, 9.86%), which suggests that the psychiatric symptoms caused by AEDs deserve attention from clinicians.

In our study, 10.6% (537/5049) of the ADRs were SARs, and 0.67% (34/5049) were SAEs. These results were supported by the results of a cross-sectional study in Iran [20]. Namazi, S. et al. [20] reported that almost all (99.24%) detected ADRs to AEDs were not serious. Among the 537 SARs, we found that rash was a common reaction to CBZ, LTG, and OXC administered as single drugs; some of the rashes were serious, including DHS and erythema multiforme. The ADRs to TPM were mainly cognitive side effects, hypoesthesia and renal calculus. The ADRs to VPA were gastrointestinal disturbances and hepatotoxicity. LEV had the fewest SARs. Our findings are not novel [6, 23, 24]; current studies have shown that rashes related to AEDs are mostly idiosyncratic reactions [22]. Godhwani, N. et al. [25] reported that non-IgE-mediated reactions, more commonly associated with aromatic AEDs, can be nonspecific rashes or severe cutaneous drug reactions (SCDRs). Moreover, recent studies have demonstrated significant associations between human leukocyte antigens (HLA) and a predisposition to ADRs [26]. Ramirez, E. et al. [27] confirmed the strong association between HLA*31:01 and CBZ-drug reactions with eosinophilia and systemic symptoms (DRESS) in Europeans. The findings of studies by Chen, C. B. et al. [28] and Chong, K. W. et al. [29] suggested that HLA-B*15:02 is significantly associated with AED-induced SCDRs in Chinese individuals. Because of the strong genetic predisposition for certain AEDs to cause serious reactions, HLA analysis before the initiation of drug therapy is advised in certain populations [25].

Furthermore, we reported that the rate of SARs after a low treatment dose (dosage/DDD ≤ 0.50) was 14.5% (*p* <  0.001). Our study also showed that more SARs occurred with multidrug treatment. The SAR rates caused by one, two and three or more AEDs were 9.9, 10.0 and 19.6%, respectively (*p* <  0.001). This result suggests that clinicians should be alert for SARs, especially when three or more AEDs are used together. These findings were supported by those of other relevant studies. In the research by Horvath, L. et al. [18], the incidences of ADRs in monotherapy, biotherapy and polytherapy were 16.4, 18.5 and 23.5%, respectively. The results of the study by Grundmann, M. et al. [30] showed that a higher number of drugs at supratherapeutic levels in combination therapy led to a 3-fold higher incidence of ADRs. Studies have reported that multiple drug therapies can increase the frequency of ADRs and teratogenic risk [31, 32]. Therefore, we suggest that the AEDs used in combination must be carefully selected [31].

## Conclusions

In summary, this study was a real-world study; therefore, the results were more consistent with the clinical situation. Our findings suggest that clinicians should pay attention to psychiatric ADRs and should be alert for SARs, especially when three or more AEDs are used together. However, the confounding factors that existed when combinations of the six AEDs were used could not be estimated. Nevertheless, our study provides important reference information to guide clinicians in the safe use of AEDs. Moreover, the definition of SARs was clarified in this study, which will help clinicians accurately use the term “severe” and improve the accuracy of the monitoring data. Finally, compared with the SR system, there was a higher quality of data in this active monitoring study; only 194 ADRs (3.8% of the total ADRs) were assessed as conditional/unclassified, with more data needed. Active surveillance might provide another method of pharmacovigilance in China.

## Additional file

Additional file 1: **Table S1.** Detailed information on 537 severe adverse reactions caused by drugs. Lists the severe adverse reactions to each drug, which may be found in the online version of this article. (DOCX 34 kb)

## Abbreviations

ADRs: Adverse drug reactions
AEDs: Antiepileptic drugs
CBZ: Carbamazepine
CFDA: China Food and Drug Administration
CNS: Central nervous system
DDD: Defined daily dose
DHS: Drug hypersensitivity syndrome
DRESS: Drug reactions with eosinophilia and systemic symptoms
ELFURS: Epilepsy Long-term Follow Up Registry Study
FAHWMU: First Affiliated Hospital of Wenzhou Medical University
HLA: Human leukocyte antigens
LEV: Levetiracetam
LTG: Lamotrigine
OXC: Oxcarbazepine
PTs: Preferred terms
RCTs: Randomized controlled trials
SAEs: Serious adverse effects
SARs: Severe adverse reactions
SCDRs: Severe cutaneous drug reactions
SOC: System organ class
SR: Spontaneous reporting
TPM: Topiramate
VPA: Valproate
WEFURD: Wenzhou Epilepsy Follow-Up Registry Database
WHO: World Health Organization
WHO-ART: WHO Adverse Reaction Terminology
WHO-UMC: WHO-Uppsala Monitoring Centre
