# Supplementary material for: Adverse drug reactions associated with six commonly used antiepileptic drugs in southern China from 2003 to 2015
Source: BMC Pharmacol Toxicol. 2019 Jan 14;20:7. doi: 10.1186/s40360-019-0285-y (PMC6332546; doi:10.1186/s40360-019-0285-y)
Supplement: Supplementary file 1 — Table S1. Detailed information on 537 severe adverse reactions caused by drugs. Lists the severe adverse reactions to each drug, which may be found in the online version of this article. (DOCX 34 kb) [file 40360_2019_285_MOESM1_ESM.docx]

Additional file 1: **Table S1.** Detailed information on 537 severe adverse reactions caused by drugs

| Drugs | Dosage/DDD | | Age when ADR occurred | | Male  (n) | Number (number of SAEs) | Severe adverse reactions |
| --- | --- | --- | --- | --- | --- | --- | --- |
|  | Median | Range | Median | Range |  |  | Preferred terms and number (number of SAEs) |
| CBZ | 0.30 | 0.10-0.90 | 28.0 | 14.0-71.0 | 29 | 56 (2) | Rash 12 (0); Leukopenia 11 (0); Dizziness 9 (0); Increased hepatic enzymes 3 (0); Nausea and vomiting 2 (0); Drug hypersensitivity syndrome 2 (2); Hypoesthesia 2 (0); Insomnia 2 (0); Diarrhea 1 (0); Ulcerative stomatitis 1 (0); Allergy 1 (0); Abnormal hepatic function 1 (0); Increased gamma-GT 1 (0); Increased alkaline phosphatase 1 (0); Muscle weakness 1 (0); Involuntary muscle contractions 1 (0); Headache 1 (0); Hallucination 1 (0); Genital ulceration 1 (0); Pruritus 1 (0); Asthenia 1 (0). |
| LEV | 0.67 | 0.33-0.67 | 37.0 | 14.0-43.0 | 7 | 8 (0) | Palpitations 1 (0); Nausea and vomiting 1 (0); Abdominal pain 1 (0); Headache 1 (0); Irritability 1 (0); Pruritus 1 (0); Asthenia 1 (0); Chest pain 1 (0). |
| LTG | 0.33 | 0.08-1.00 | 25.0 | 12.0-65.0 | 12 | 39 (2) | Rash 24 (0); Dizziness 2 (0); Pruritus 2 (0); Erythema multiforme 1 (1); Multiple malformations 1 (1); Leukopenia 1 (0); Nausea and vomiting 1 (0); Viral infection 1 (0); Muscle weakness 1 (0); Hypoesthesia 1 (0); Insomnia 1 (0); Abnormal sexual function 1 (0); Menstrual disorder 1 (0); Increased lacrimation 1 (0). |
| OXC | 0.60 | 0.30-1.80 | 33.0 | 10.0--75.0 | 17 | 47 (1) | Rash 18 (0); Dizziness 7 (0); Somnolence 3 (0); Pruritus 2 (0); Nausea and vomiting 2 (0); Asthenia 2 (0); Drug hypersensitivity syndrome 1 (1); Leukopenia 1 (0); Arrhythmia 1 (0); Diarrhea 1 (0); Constipation 1 (0); Abdominal pain 1 (0); Decreased weight 1 (0); Tinnitus 1 (0); Tremor 1 (0); Ataxia 1 (0); Headache 1 (0); Anorexia 1 (0); Localized inflammation 1 (0). |
| TPM | 0.33 | 0.08-1.17 | 28.0 | 8.0-58.0 | 21 | 46 (9) | Amnesia 6 (1); Hypoesthesia 6 (0); Renal calculus 5 (5); Dizziness 4 (0); Decreased weight 3 (0); Stupor 2 (0); Depression 2 (0); Anorexia 2 (0); Congenital spinal tumor 1 (1); Cognitive disorder 1 (1); Exfoliative dermatitis 1 (1); Nausea and vomiting 1 (0); Increased hepatic enzymes 1 (0); Back pain 1 (0); Aphasia 1 (0); Tremor 1 (0); Somnolence 1 (0); Anxiety 1 (0); Insomnia 1 (0); Abnormal thoughts 1 (0); Abnormal sexual function 1 (0); Menstrual disorder 1 (0); Rash 1 (0); Fatigue 1 (0). |
| VPA | 0.67 | 0.33-1.67 | 25.0 | 11.0-57.0 | 21 | 56 (3) | Nausea and vomiting 10 (0); Increased hepatic enzymes 9 (0); Menstrual disorder 3 (0); Tremor 3 (0); Increased weight 3 (0); Rash 3 (0); Amnesia 2 (0); Leukopenia 2 (0); Dyspepsia 2 (0); Asthenia 2 (0); Somnolence 2 (0); Thrombocytopenia 1 (1); Abnormal vision 1 (1); Constipation 1 (0); Eating disorder 1 (0); Drug hypersensitivity syndrome 1 (1); Hepatitis 1 (0); Muscle weakness 1 (0); Myalgia 1 (0); Dizziness 1 (0); Anxiety 1 (0); Insomnia 1 (0); Ovarian disorder 1 (0); Larynx pain 1 (0); Alopecia 1 (0); Fatigue 1 (0). |
| CBZ+LEV | 1.27 | 0.63-1.27 | 24.0 | 17.0-30.0 | 2 | 4 (0) | Leukopenia 1 (0); Gamma-GT increased 1 (0); Muscle weakness 1 (0); Dizziness 1 (0). |
| CBZ+LTG | 1.00 | 0.50-1.20 | 22.0 | 18.0-65.0 | 4 | 7 (0) | Nausea and vomiting 1 (0); Allergy 1 (0); Speech disorder 1 (0); Dizziness 1 (0); Somnolence 1 (0); Rash 1 (0); Vascular disorder 1 (0). |
| CBZ+TPM | 1.25 | 0.43-1.63 | 21.0 | 19.0-32.0 | 2 | 6 (1) | Amnesia 2 (0); Renal calculus 1 (1); Increased hepatic enzymes 1 (0); Aphasia 1 (0); Stupor 1 (0). |
| CBZ+VPA | 1.23 | 0.43-1.50 | 24.0 | 14.0-63.0 | 18 | 35 (2) | Nausea and vomiting 6 (0); Dizziness 3 (0); Leukopenia 2 (0); Dyspepsia 2 (0); Increased weight 2 (0); Increased hepatic enzymes 2 (0); Tremor 2 (0); Somnolence 2 (0); Amnesia 2 (0); Rash 2 (0); Gastrointestinal malformation 1 (1); Fever 1 (1); Neutropenia 1 (0); Increased gamma-GT 1 (0); Anorexia 1 (0); Menstrual disorder 1 (0); Alopecia 1 (0); Increased sweating 1 (0); Dermographia 1 (0); Diplopia 1 (0). |
| LEV+LTG | 0.92 | / | 63.0 | / | 0 | 1 (0) | Pharyngitis 1 (0). |
| LEV+OXC | 1.15 | 0.78-1.63 | 37.0 | 19.0-50.0 | 3 | 10 (1) | Somnolence 3 (0); Leukopenia 2 (0); Increased hepatic enzymes 1 (0); Rhabdomyolysis 1 (1); Dizziness 1 (0); Rash 1 (0); Pruritus 1 (0). |
| LEV+TPM | 1.33 | 0.83-1.33 | 30.0 | 27.0-30.0 | 0 | 4 (0) | Decreased weight 1 (0); Headache 1 (0); Sleep disorder 1 (0); Fatigue 1 (0). |
| LEV+VPA | 0.92 | 0.67-1.33 | 43.0 | 17.0-67.0 | 6 | 18 (3) | Irritability 3 (0); Pancreatitis 2 (2); Tremor 2 (0); Psychosis 1 (1); Leukopenia 1 (0); Nausea and vomiting 1 (0); Diarrhea 1 (0); Face edema 1 (0); Hypoesthesia 1 (0); Somnolence 1 (0); Insomnia 1 (0); Dreaming abnormal 1 (0); Menstrual disorder 1 (0); Fatigue 1 (0). |
| LTG+OXC | 0.93 | / | 17.0 | / | 1 | 1 (0) | Fatigue 1 (0). |
| LTG+TPM | 0.92 | 0.33-1.33 | 30.0 | 23.0-57.0 | 1 | 8 (2) | Rash 2 (0); Congenital hearing disorder 1 (1); Anemia 1 (1); Decreased weight 1 (0); Polyneuropathy 1 (0); Depression 1 (0); Anxiety 1 (0). |
| LTG+VPA | 0.92 | 0.33-1.67 | 39.0 | 13.0-67.0 | 20 | 70 (4) | Rash 24 (0); Tremor 8 (0); Nausea and vomiting 5 (0); Dizziness 5 (0); Increased hepatic enzymes 3 (0); Pruritus 3 (0); Alopecia 3 (0); Exfoliative dermatitis 2 (2); Diarrhea 2 (0); Erythematous rash 1 (1); Abnormal vision 1 (1); Dyspepsia 1 (0); Increased weight 1 (0); Allergy 1 (0); Muscle weakness 1 (0); Headache 1 (0); Somnolence 1 (0); Anorexia 1 (0); Agitation 1 (0); Anxiety 1 (0); Insomnia 1 (0); Menstrual disorder 1 (0); Dry skin 1 (0); Diplopia 1 (0). |
| OXC+TPM | 1.10 | 0.63-2.02 | 29.0 | 15.0-62.0 | 8 | 17 (2) | Renal calculus 2 (2); Nausea and vomiting 2 (0); Dizziness 2 (0); Decreased weight 1 (0); Muscle weakness 1 (0); Cognitive disorder 1 (0); Headache 1 (0); Anorexia 1 (0); Insomnia 1 (0); Abnormal thoughts 1 (0); Amnesia 1 (0); Pharyngitis 1 (0); Rash 1 (0); Asthenia 1 (0). |
| OXC+VPA | 1.10 | 0.63-2.13 | 27.0 | 15.0-54.0 | 6 | 16 (0) | Rash 4 (0); Nausea and vomiting 2 (0); Somnolence 2 (0); Leukopenia 1 (0); Abdominal pain 1 (0); Hyperammonemia 1 (0); Tinnitus 1 (0); Increased hepatic enzymes 1 (0); Dizziness 1 (0); Anxiety 1 (0); Menstrual disorder 1 (0). |
| TPM+VPA | 1.17 | 0.42-1.33 | 19.0 | 11.0-41.0 | 7 | 17 (0) | Amnesia 3 (0); Diarrhea 2 (0); Increased hepatic enzymes 2 (0); Nausea and vomiting 1 (0); Abdominal pain 1 (0); Enlarged abdomen 1 (0); Decreased weight 1 (0); Hypoesthesia 1 (0); Tremor 1 (0); Anorexia 1 (0); Abnormal dreaming 1 (0); Irritability 1 (0); Alopecia 1 (0). |
| CBZ+LTG+LEV | 1.67 | / | 15.0 | / | 1 | 1 (0) | Irritability 1 (0). |
| CBZ+TPM+LEV | 1.67 | 1.33-2.00 | 25.0 | 20.0-29.0 | 2 | 3 (0) | Tremor 1 (0); Irritability 1 (0); Rash 1 (0). |
| CBZ+VPA+LEV | 1.54 | 1.23-2.00 | 33.0 | 24.0-38.0 | 4 | 6 (0) | Dry mouth 1 (0); Diarrhea 1 (0); Decreased weight 1 (0); Increased hepatic enzymes 1 (0); Somnolence 1 (0); Menstrual disorder 1 (0). |
| CBZ+VPA+LTG | 1.20 | 1.03-1.63 | 22.0 | 14.0-60.0 | 2 | 7 (1) | Rash 2 (0); Ovarian disorder 1 (1); Leukopenia 1 (0); Increased alkaline phosphatase 1 (0); Abnormal coordination 1 (0); Somnolence 1 (0). |
| CBZ+VPA+TPM | 1.30 | 0.93-1.67 | 18.0 | 16.0-37.0 | 1 | 5 (0) | Nausea and vomiting 4 (0); Dizziness 1 (0). |
| LEV+LTG+OXC | 1.43 | 1.43-1.43 | 39.0 | 39.0-39.0 | 2 | 2 (0) | Nausea and vomiting 1 (0); Dizziness 1 (0). |
| LEV+LTG+TPM | 1.67 | 1.00-2.00 | 29.0 | 28.0-56.0 | 3 | 4 (0) | Anorexia 1 (0); Agitation 1 (0); Insomnia 1 (0); Rash 1 (0). |
| LEV+OXC+TPM | 1.59 | 1.27-1.90 | 29.0 | 27.0-31.0 | 0 | 4 (1) | Abnormal behavior 1 (1); Hallucination 1 (0); Somnolence 1 (0); Asthenia 1 (0). |
| LEV+VPA+LTG | 1.33 | 0.83-1.50 | 33.0 | 22.0-36.0 | 2 | 5 (0) | Speech disorder 1 (0); Somnolence 1 (0); Rash 1 (0); Alopecia 1 (0); Fever 1 (0). |
| LEV+VPA+OXC | 1.90 | 1.90-2.00 | 25.0 | 25.0-26.0 | 1 | 2 (0) | Increased weight 1 (0); Irritability 1 (0). |
| LTG+CBZ+TPM | 0.83 | 0.67-1.00 | 36.0 | 25.0-47.0 | 1 | 2 (0) | Irritability 1 (0); Rash 1 (0). |
| LTG+VPA+OXC | 1.27 | 0.80-2.00 | 24.0 | 18.0-54.0 | 3 | 7 (0) | Rash 3 (0); Nausea and vomiting 1 (0); Muscle weakness 1 (0); Abnormal sexual function 1 (0); Alopecia 1 (0). |
| LTG+VPA+TPM | 1.33 | 1.17-2.05 | 26.0 | 11.0-54.0 | 6 | 10 (0) | Rash 2 (0); Dyspepsia 2 (0); Decreased weight 1 (0); Abnormal hepatic function 1 (0); Hepatic enzymes increased 1 (0); Cognitive disorder 1 (0); Speech disorder 1 (0); Amnesia 1 (0). |
| OXC+CBZ+TPM | 1.32 | 0.93-1.70 | 26.0 | 26.0-26.0 | 2 | 2 (0) | Dizziness 1 (0); Asthenia 1 (0). |
| OXC+VPA+TPM | 1.45 | 0.90-1.78 | 32.0 | 22.0-52.0 | 2 | 3 (0) | Speech disorder 1 (0); Somnolence 1 (0); Periorbital edema 1 (0). |
| CBZ+TPM+VPA+LTG | 1.62 | 1.23-2.00 | 24.0 | 22.0-25.0 | 1 | 2 (0) | Coughing 1 (0); Diplopia 1 (0). |
| LEV+CBZ+VPA+OXC | 1.67 | 1.67-1.67 | 61.0 | 61.0-61.0 | 0 | 2 (0) | Dyspepsia 1 (0); Headache 1 (0). |
| LTG+VPA+OXC+LEV | 1.15 | / | 39.0 | / | 0 | 1 (0) | Pruritus 1 (0). |
| OXC+VPA+TPM+LEV | 1.80 | 0.90-1.80 | 47.0 | 47.0-47.0 | 0 | 3 (0) | Back pain 1 (0); Somnolence 1 (0); Micturition frequency 1 (0). |
|  | | | | | | | |

Note: ADR, adverse drug reaction; DDD, defined daily dose; SAEs, serious adverse effects; CBZ, carbamazepine; VPA, valproate; LTG, lamotrigine; OXC, oxcarbazepine; TPM, topiramate; LEV, levetiracetam.
